# Spatial distribution and feeding substrate of butterflyfishes (family Chaetodontidae) on an Okinawan coral reef

**DOI:** 10.7717/peerj.9666

**Published:** 2020-08-04

**Authors:** Atsushi Nanami

**Affiliations:** Research Center for Sub-tropical Fisheries, Seikai National Fisheries Research Institute, Japan Fisheries Research and Education Agency, Ishigaki, Okinawa, Japan

**Keywords:** Butterflyfishes, Spatial distribution, Feeding behavior, Coral reefs, Substrate diversity, Coral polyp feeder, Benthic animal feeder

## Abstract

Coral reefs support diverse communities, and relationships among organisms within these communities are quite complex. Among the relationships, clarifying the habitat association and foraging substrate selection relative to habitat characteristics is of central importance to ecology since these two aspects are the fundamentals for survival and growth of organisms. The aims of the present study were to investigate the spatial distribution and feeding substrate selection of 14 species of butterflyfishes on an Okinawan coral reef in Japan. Species-specific spatial distributions varied with habitat characteristics (e.g., encrusting corals, massive corals, branching *Acropora* and rock). For feeding substrates, seven species of obligate coral polyp feeders exhibited significant positive selectivity for tabular *Acropora*, corymbose *Acropora*, encrusting corals and massive corals but significant negative selectivity for dead corals, coral rubble and rock. Among six species of facultative coral polyp feeders, two species exhibited significant positive selectivity for encrusting corals and massive corals, and one species showed significant positive selectivity for dead corals as feeding substrates. In contrast, three species exhibited no significant positive selectivity for any feeding substrates. A similar result was observed for one non-coralline invertebrate feeder. Among the 14 species, 12 species showed a relatively close relationship between spatial distribution and feeding substrates but the remaining two species did not. The present study is the first study to elucidate species-specific spatial distributions and feeding substrate selection of butterflyfishes on an Okinawan coral reef. The results of the present study suggest that diverse substrates, including various types of living corals (especially encrusting corals, massive corals, tabular *Acropora*, corymbose *Acropora* and branching *Acropora*) and non-coralline substrates (rock) are the primary determinants of spatial distributions and feeding sites. Thus, diverse substrates are important for maintaining high species diversity of butterflyfishes and changes of the substrates would likely change the spatial patterns and foraging behavior, although species-specific responses may be found, depending on their species-specific dependence on vulnerable substrates.

## Introduction

Coral reefs support diverse species, and relationships among these marine organisms are complex. Such relationships include competition (e.g.,  Robertson, 1980; Nanami, 2018), predation (e.g.,  Hixon, 1991; Hixon & Carr, 1997), foraging (e.g.,  Nanami & Shimose, 2013; Smith et al., 2018) and habitat association (e.g.,  Meekan, Steven & Fortin, 1995; Beans, Jones & Caley, 2002; Eurich, McCormick & Jones, 2018). Among the relationships, identifying spatial distributions of species relative to habitat characteristics is of central importance to ecology. Knowledge of habitat associations provides a foundation for the interpretation of interactions between species and environmental characteristics. This knowledge also assists in the identification of objectives for the preservation of biodiversity, since habitat conservation is essential for the protection of focal target species.

Coral reefs exhibit diverse habitat characteristics comprising both benthic organisms and nonliving substrates. Numerous studies show that living corals are important determinants of spatial distribution for many fish species, since living corals provide food and shelter (e.g.,  Williams, 1991; Munday, Jones & Caley, 1997; Nanami et al., 2005; Pratchett, Graham & Cole, 2013). In addition, abiotic factors (e.g., wave exposure, water depth, and topographic complexity) also have significant effects on fish spatial distribution (e.g.,  Luckhurst & Luckhurst, 1978; McCormick, 1994; Fulton, Bellwood & Wainwright, 2001; Fulton & Bellwood, 2002).

Clarifying the foraging behavior of marine organisms is also essential since foraging is the fundamental aspect for survival and growth. Many previous studies have identified food items of various coral reef fish species (e.g.,  Hiatt & Strasburg, 1960; Sano, Shimizu & Nose, 1984; Choat, Clements & Robbins, 2002; Marnane & Bellwood, 2002; Nagelkerken et al., 2009; Nanami & Shimose, 2013; Nanami, 2018). Ecological information on feeding substrates, i.e., substrates from which organisms take food items, have been also shown (Cole, Pratchett & Jones, 2008; Rotjan & Lewis, 2008). Substrates in coral reefs are diverse, including many species of corals, soft corals, macroalgae, other benthic organisms that live on these major substrate groups, rock, coral rubble and sand. Thus, the foraging substrates of coral reef fishes are also highly diverse. Some previous studies have recorded substrates from which reef fish forage using direct underwater observations. For example, Nanami & Yamada (2008) reported that a predatory lutjanid species preferentially foraged over branching and dead corals, largely due to the presence of small prey species that shelter in these substrates. Kramer, Bellwood & Bellwood (2016) revealed significant positive use of dead corals, coral rubble and epilithic algal matrices as foraging microhabitats of wrasses which an earlier report had shown to have greater densities of benthic crustaceans as compared to with living corals (Kramer, Bellwood & Bellwood, 2014). In contrast, some herbivores, such as parrotfishes and rabbitfishes, feed mainly on epilithic algal matrices, macroalgae and sea sponges attached to rock (Clements et al., 2017; Smith et al., 2018; Nanami, 2018). Thus, the use of foraging substrate is diverse and species-specific, and clarifying such diversity of foraging behavior would improve our ecological understanding of coral reef ecosystems.

The butterflyfishes (family Chaetodontidae) include four dietary groups: obligate coral polyp feeders, facultative coral polyp feeders, non-coralline invertebrate feeders, and zooplankton feeders (Sano, 1989; Cole, Pratchett & Jones, 2008). Previous studies have determined spatial distributions of butterflyfishes in relation to habitat characteristics (e.g.,  Fowler, 1990; Alwany, El-Etreby & Hanafy, 2007; Pratchett & Berumen, 2008; Emslie et al., 2010; Khalaf & Abdallah, 2014; Pratchett, Graham & Cole, 2013; Pratchett et al., 2014). Most of these studies have revealed species-specific differences in density among reef zones. The feeding behavior and substrate selection for foraging have also been identified. Some previous studies have indicated clear differences in substrate selection for foraging among multiple species (e.g.,  Tricas, 1989; Nagelkerken et al., 2009; Pratchett et al., 2014; Liedke et al., 2016). In addition, the substrate selection for foraging is affected by substrate availability (e.g.,  Berumen, Pratchett & McCormick, 2005; Zambre & Arthur, 2018). Geographical variation in feeding substrates and the impact of coral community degradation on the feeding behavior have also been described and discussed. Lawton et al. (2012) demonstrated that four species of obligate coral polyp feeders had region-specific substrate selectivity due to different substrate availability among five geographical regions (Papua New Guinea, New Caledonia, French Polynesia, Lizard Island and Heron Island). Pratchett & Berumen (2008) have discussed that loss of live coral cover due to global climate change would cause negative impact of foraging behavior, especially for obligate coral polyp feeders.

Since some of butterflyfishes consist of coral polyp feeders, their habitat association might be closely related with the preferred foraging substrates. However, studies clarifying the degree of linking between habitat associations and feeding substrates are still limited (but see Pratchett & Berumen, 2008). Pratchett & Berumen (2008) have suggested that it is unclear whether the restricted distributions of butterflyfishes are caused by dietary specialization. Since most previous studies have shown zonational patterns of butterflyfishes, clarifying the relationship between substrates as habitat as well as substrates as foraging site would provide a more comprehensive understanding about the ecology of butterflyfishes. Such knowledge would also improve out capacity to predict the effect of coral community degradation on butterflyfish community.

One of the recent threats which cause negative impact to species diversity of coral reefs is coral bleaching. Recently, severe coral bleaching occurred in 1998, 2002, 2003, 2007 and 2016 in Okinawan region (Shibuno et al., 1999; Loya et al., 2001; Ministry of the Environment & Japanese Coral Reef Society, 2004; Sano, 2004; Okamoto, Nojima & Furushima, 2007; Kayanne, Suzuki & Liu, 2017). The coverage of acroporid corals decreased in >90% due to the coral bleaching and subsequent death ( Shibuno et al., 1999; Sano, 2004). Consequently, most of fish species that categorized as coral polyp feeders (e.g., some species of butterflyfishes, wrasses and leatherjackets) disappeared after the coral bleaching (Sano, 2004). Thus, it is suggested that coral bleaching causes significant decline of species diversity in Okinawan region. It is also suggested that rising seawater temperature by global warming would increase the frequency of coral bleaching in Okinawan region in the future (Yara et al., 2009; Yara et al., 2014). Nevertheless, species diversity of coral reefs in the Okinawan region is very high and protection of such high species diversity is important (Ministry of the Environment, Japanese Coral Reef Society, 2004). Such protection requires understanding the spatial distribution as well as foraging behavior of focal marine organisms in relation to environmental characteristics. Butterflyfishes are a component of Okinawan coral reef communities, but ecological studies on spatial distribution and feeding behavior have not been conducted. In addition, there has not been sufficient study of the degree of linking between habitat associations and feeding substrates.

The aim of the present study was to investigate the spatial distribution and feeding substrate selection for 14 species of butterflyfishes on an Okinawan coral reef. Specifically, the goals were to elucidate (1) the relationships between the spatial distribution and habitat characteristics, (2) the species-specific substrate selections for foraging and (3) the relationship between habitat association and feeding substrates. The potential impacts of coral community degradation induced by coral bleaching on the spatial distribution and feeding behavior of butterflyfishes is also considered, i.e., which butterflyfish species are more vulnerable or resilient for coral bleaching in terms of habitat and food. Since some previous studies have shown regional difference in spatial distribution and foraging behavior of butterflyfishes, the results of the present study would provide new additional insights about ecology of butterflyfishes.

## Materials and Methods

### Study species

Although 45 butterflyfish species were recorded in Okinawan region (Nakabo, 2000), 26 species were found in the study site (Table S1). Among the 26 species, 14 species were examined in the present study, since these species showed higher density. The 14 species include three dietary groups (Table S2): seven species of obligate coral polyp feeders (*C. baronessa*, *C. bennetti*, *C. lunulatus*, *C. ornatissimus*, *C. plebeius*, *C. punctatofasciatus*, and *C. trifascialis*), six species of facultative coral polyp feeders (*C. argentatus*, *C. auriga*, *C. citrinellus*, *C. ephippium*, *C. kleinii*, and *C. vagabundus*) and one non-coralline invertebrate feeder (*Forcipiger flavissimus*).

### Spatial distribution

**Figure 1:**
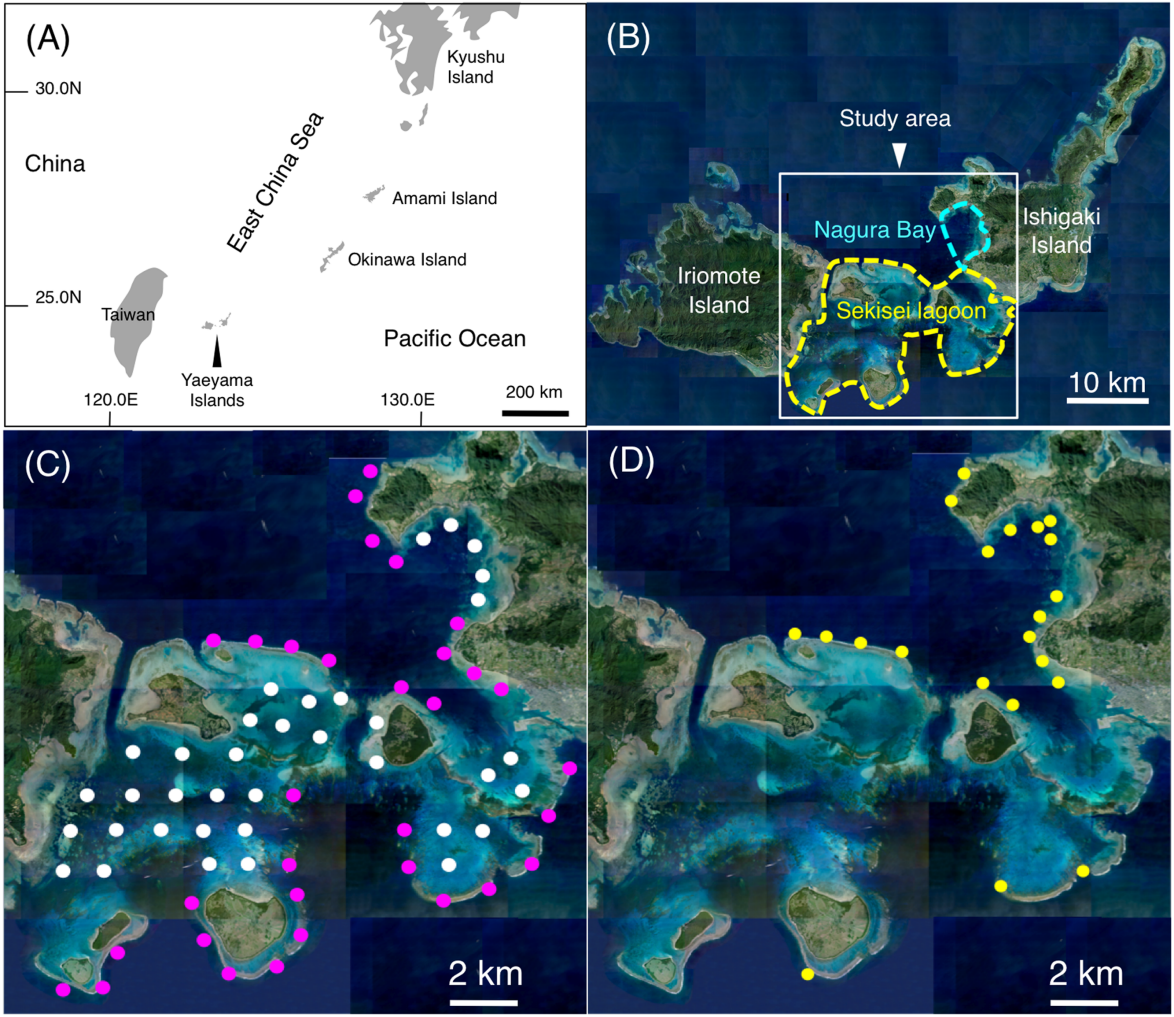
Study site. The maps show the location of the Yaeyama Islands (A), Sekisei Lagoon and Nagura Bay (B), the 68 study sites used for underwater observations of spatial distributions (C), and the 21 sites used for underwater observations of feeding behavior (D). In (B), yellow and sky-blue dashed lines indicate the boundaries of Sekisei Lagoon and Nagura Bay, respectively. In (C), magenta symbols and white symbols indicate the sites located in exposed and inner reefs, respectively. In (D), yellow symbols indicate the sites used for underwater observations of feeding behavior. Photo credit: International Coral Reef Research and Monitoring Center.

Underwater visual observations were conducted at Sekisei Lagoon and Nagura Bay in the Yaeyama Islands, Okinawa, Japan (Figs. 1A and 1B). In order to include almost the total area in the study site, multiple sites with inter-site distance was about 2 km were established. As a result, 68 study sites including 32 sites on exposed reefs and 36 sites on inner reefs were established (Fig. 1C). The observations were conducted between June 2016 and February 2017.

Underwater visual survey was conducted in accordance with Nanami (2018). In brief, at each site, one 20-min observation was conducted along a 5-m wide transect using SCUBA. A portable GPS receiver was used to measure the length of each transect. The average distances covered were 343.1 ± 43.9 m [mean ± standard deviation (SD), minimum length = 233 m maximum length = 439 m]. On each transect, the number of individuals and total length of butterflyfishes were recorded. Then, density of each of the 14 butterflyfish species (number of individuals per 500 m^2^) was obtained by using above-mentioned data (number of individuals and length of 20-min transect) for each site. Depth profiles were obtained by using a diving computer. The interval of depth data recording was 30-s, providing 40 depth values for each site (two depth points per1 min × 20 min). Then, the 40 depth profiles were averaged for each site. The water depths ranged from 2.6 m to 12.4 m (average ± SD = 7.66 ± 1.95 m).

### Spatial variation in substrates

The substrate characteristics at each site were recorded by a digital camera. A digital camera was attached to the data collection board using PVC pipes (Fig. S2 ), making it possible to collect digital images and fish data simultaneously. The distance between the video camera and substrate was about 2 m. Static images were obtained at 10-s intervals, providing 121 static images per 20-min transect. For each image, the substrate occupying the center of the image was identified. Substrates were divided into 17 categories for analysis modified from Nanami (2018): (1) branching *Acropora* (e.g., *A. formosa*), (2) bottlebrush *Acropora* (e.g., *A. carduus*), (3) tabular *Acropora* (e.g., *A. hyacinthus*), (4) corymbose *Acropora* (e.g., *Acropora nasuta*), (5) *Pocillopora*, (6) branching corals other than genera *Acropora* and *Pocillopora*. (e.g., branching *Montipora* and *Porites*), (7) encrusting corals (e.g., encrusting Pectinidae, Faviidae, Poritidae, *Echinophyllia*, *Hydnophora*, *Scapophyllia*, and *Montipora*), (8) leafy corals (e.g., *Echinopora lamellosa*), (9) massive corals (e.g., massive Poritidae, Oculinidae, Faviidae, and Mussidae), (10) mushroom corals, (11) other living corals (e.g., *Pavona* and *Goniopora*), (12) soft corals, (13) dead corals, (14) coral rubble, (15) rock (calcium carbonate substratum with lower substrate complexity), (16) sand, and (17) macroalgae (e.g., *Padina minor* and *Sargassum* spp.). By using the total number of images for each substrate category, the percent cover for the each of the 17 categories was calculated.

### Spatial distribution analysis

To test whether fish density or substrate coverage differed between exposed and inner reefs, a generalized linear mixed model (GLMM) with zero-inflated negative binominal error distribution were performed for each fish species and each substrate. In the analysis, glmmTMB package in R statistical computing language were used (R Core Team, 2017) with site as a random term and reefs as the fixed term. Bonferroni correction was applied to test the significant difference. The relationship between the spatial distribution of the 14 butterflyfish species and environmental characteristics (17 substrates plus depth) was analyzed using redundancy analysis (RDA) using CANOCO software (Ter Braak & Smilauer, 2002). Since time-transect (a 20-min swimming at each site) was applied, one density data and one substrate coverage data were obtained at each site. Prior to the analysis, the fish density data were square-root transformed. Forward selection was applied to identify the environmental characteristics which have significant effects on the spatial distributions of the 14 butterflyfish species.

### Feeding behavior

To elucidate the feeding behavior, underwater visual observations were conducted at 21 study sites between June 2016 and February 2020 (Fig. 1D). In order to collect the data of feeding behavior effectively, the 21 sites were selected due to relatively greater density of the 14 species.

Five-minute focal observations of individual fish were made by an observer following at a distance of about 3–5 m. The observer recorded the number of bites on each substrate type and estimated the total length of the individual. At the end of the observation period, a new focal individual was chosen, taking care not to choose the same individual. Most individuals did not change their behavior and continued feeding as the observer approached. However, if individuals changed behavior (e.g., fleeing from the observer) when approached, the observation was aborted. To examine the substrate characteristics, 50 quadrats (50 × 50 cm) were haphazardly placed at least 10 m apart in each site. Substrates within quadrats were photographed using a digital camera. In the laboratory, the photographs of each 50 × 50 cm quadrat were divided into 100 subsections (5 × 5 cm) providing totally 5,000 subsections were obtained at each study site. Then, the above-mentioned 17 categories of substrates in each subsection were identified. For each subsection, only the predominant substrate (>50% in the subsection) was recorded. Finally, the average coverage of each substrate was calculated by dividing the number of subsections containing that substrate by the total number of subsections.

### Feeding behavior analysis

For each species, the proportion of bites on a given substrate was calculated for each site and then an average proportion over all sites was calculated. Shannon’s diversity index was applied to assess substrate use diversity after calculating the proportion of all bites for each substrate:

\begin{eqnarray*}{H}^{{^{\prime}}}=-\sum {p}_{i}{\mathrm{log}}_{\mathrm{e}}({p}_{i}) \end{eqnarray*}

where *H’* is the diversity index, and *p*_*i*_ is the proportion of bites on the *i* th substrate. To evaluate what types of substrates were used positively or negatively in relation to their availability, Strauss’ linear resource index was used (Strauss, 1979; Smith et al., 2018):

\begin{eqnarray*}{L}_{i}={r}_{i}-{P}_{i} \end{eqnarray*}

where *L*_*i*_ is Strauss’ linear resource index for the *i* th substrate, *r*_*i*_ is the proportion of bites on the *i* th substrate, and *P*_*i*_ is the proportion of the *i* th substrate in the environment. The indices were calculated for each site separately and then the average *L*_*i*_ value with 95% confidence interval (CI) was obtained for each substrate. The 95% CI was calculated as:

\begin{eqnarray*}95\text{%} {\mathrm{CI}}_{i}=t\times \mathrm{standard~ error~ of} {L}_{i} \end{eqnarray*}

where 95% CI_*i*_ is the 95% confidence interval for the *i* th substrate, and *t* is the *t*-statistic with Bonferroni correction (*p* = 0.05/17). *L*_*i*_ ± 95% CI_*i*_ >1 indicates a significant positive use of the *i* th substrate, and <1 indicates a significant negative use. *L*_*i*_ ± 95% CI_*i*_, including 0 indicates no significant selectivity for the *i* th substrate. Individuals for which the total number of bites was less than 10 were omitted from the analysis.

To summarize species-specific differences in feeding behavior, a principal component analysis (PCA) was conducted based on the numbers of bites and average *L*_*i*_ values for all substrates.

## Results

### Spatial distributions

**Figure 2:**
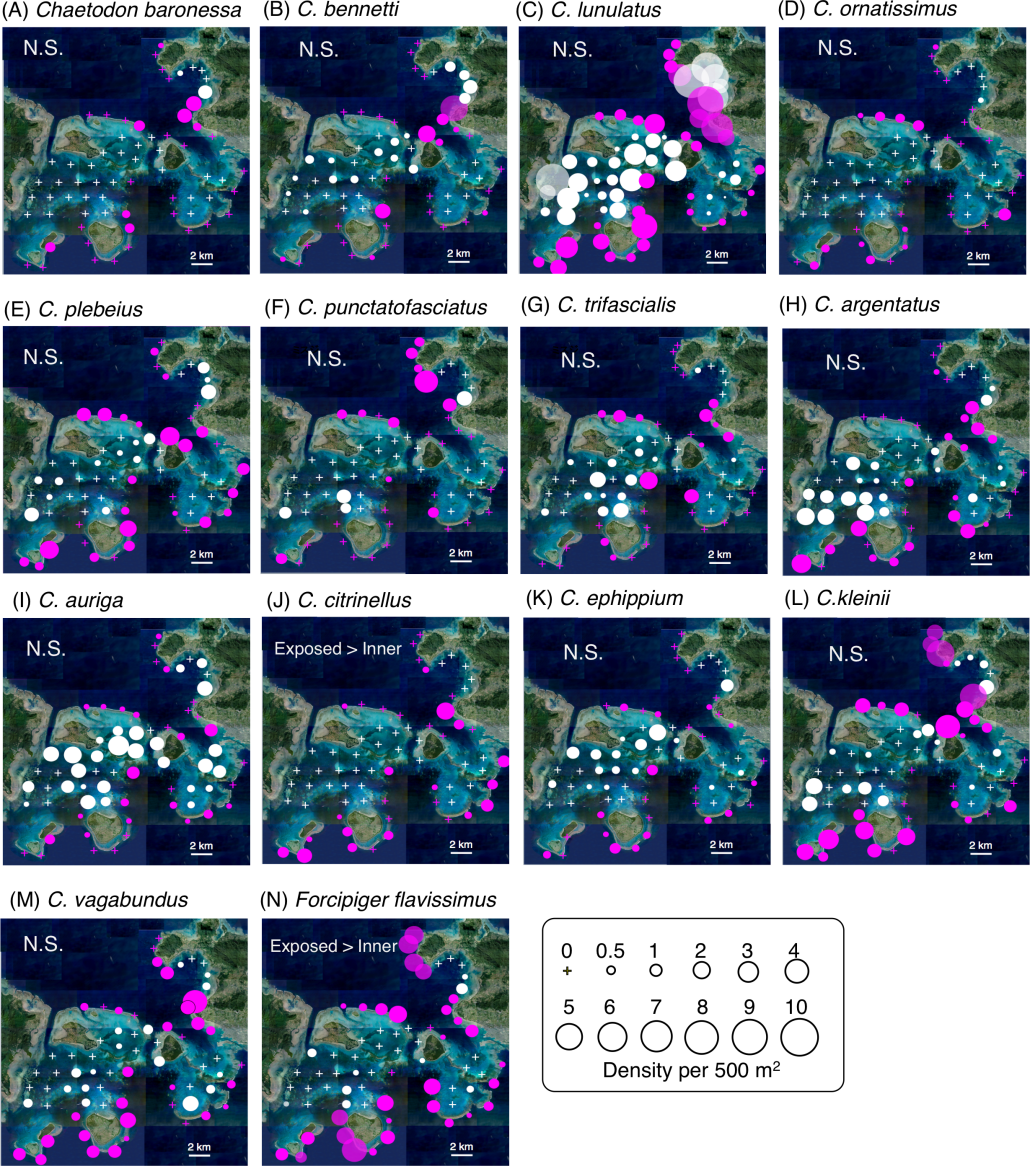
Spatial distributions of the 14 butterflyfish species at the 68 study sites in which yellow circles indicate fish density. The circle size represent the density. Magenta symbols and white symbols indicate the sites located in exposed and inner reefs, respectively. Crosses indicate no individuals were found at the study site. (A–F): obligate coral polyp feeders, (H–M): facultative coral polyp feeders, (N): the non-coralline invertebrate feeder. Each panel also shows whether or not the difference in density between exposed and inner reefs was statistically significant based on a GLMM (N.S., non-significant, see also Tables S3 for details about results of GLMM). Photo credit: International Coral Reef Research and Monitoring Center.

Among the 14 species, *C. lunulatus* appeared to be the most abundant overall regardless of habitat, and *C. baronessa* and *C. ornatissimus* the least abundant (Table S1). Significantly greater densities were found on exposed than on inner reefs for two species (*C. citrinellus* and *F. flavissimus*: Fig. 2, Table S3). The remaining twelve species did not differ significantly between exposed and inner reefs. On exposed reefs, there was significantly higher cover of encrusting corals and rock. On inner reefs there was significantly greater coverage of dead corals, coral rubble and sand (Fig. S1, Table S4).

**Figure 3:**
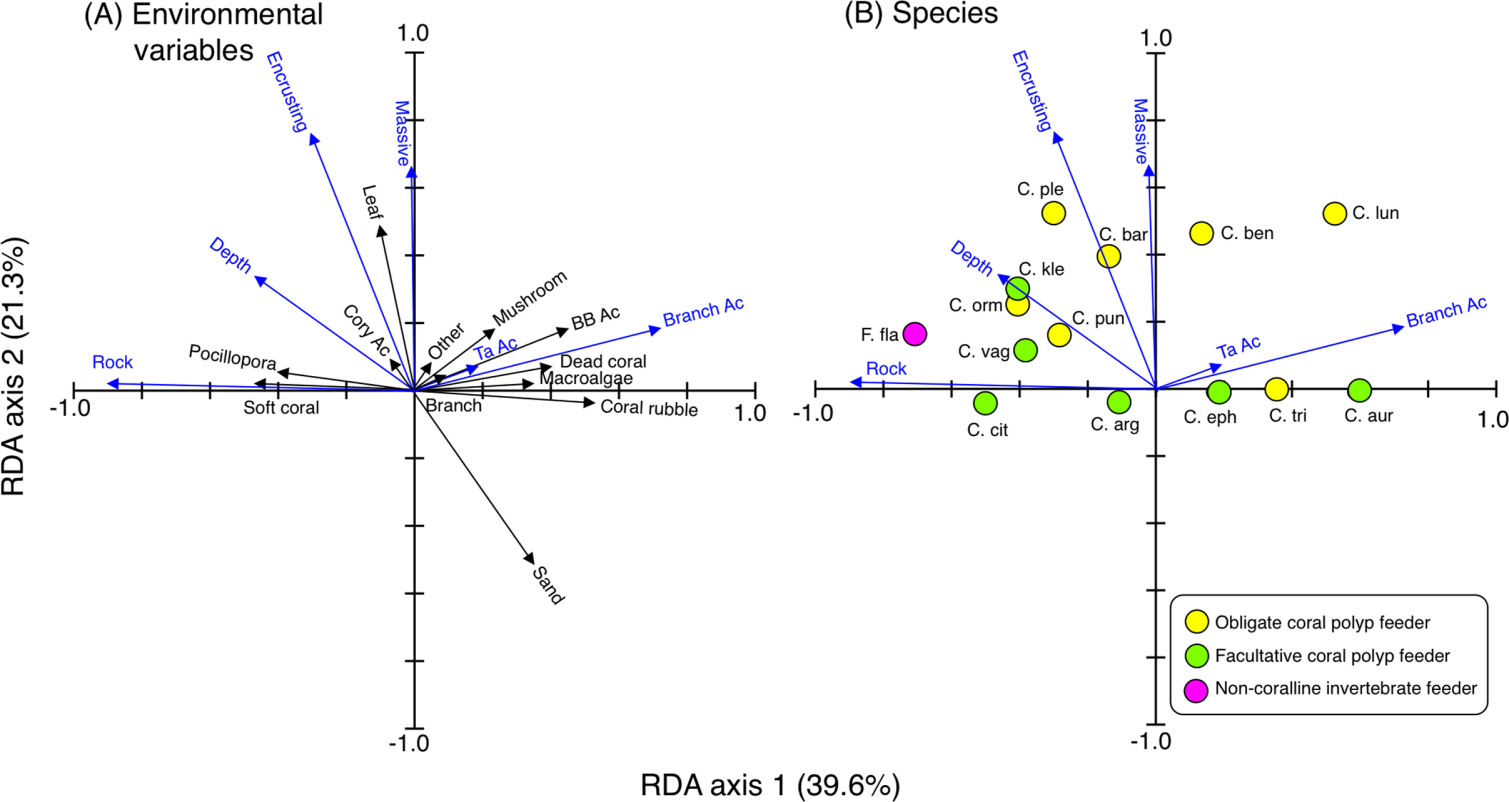
Results of the redundancy analysis (RDA) to explain the relationship between the spatial distribution of the 14 butterflyfish species and environmental characteristics, showing the vectors of environmental variables (A) and species score (B). Eighteen vectors of environmental variables (17 substrates plus depth) were shown and environmental variables that had a significant effect on spatial distributions are presented as blue vectors (A). Only those environmental variables that exhibited a significant effect are presented in (B). In (A) and (B), some types of coral are represented as abbreviations (BB Ac: bottlebrush *Acropora*, Branch Ac: branching *Acropora*, Cory Ac: corymbose *Acropora*, TA Ac: tabular *Acropora*,). In (B), species names are represented as abbreviations (C. arg: *C. argentatus*, C. aur: *C. auriga*, C. bar: *C. baronessa*, C. ben: *C. bennetti*, C. cit: *C. citrinellus*, C. eph: *C. ephippium*, C. kle: *C. kleinii,* C. lun: *C. lunulatus*, C. orn: *C. ornatissimus,* C. ple: *C. plebeius*, C. pun: *C. punctatofasciatus*, C. tri: *C. trifascialis*, C. vag: *C. vagabundus*, F. fla: *Forcipiger flavissimus*). In (B), yellow, green and magenta symbols indicate obligate coral polyp feeder, facultative coral polyp feeder and non-coralline invertebrate feeder, respectively.

RDA revealed the species-specific spatial distributions among the 14 species (Fig. 3). Four species (*C. argentatus, C. vagabundus, C. citrinellus* and *F. flavissimus*) were found at sites with greater coverage of rock, soft corals and *Pocillopora.* Three species (*C. punctatofasciatus, C. kleinii* and *C. ornatissimus*) were found at sites with greater coverage of rock, encrusting corals, massive corals, leafy corals, and greater depth. Three species (*C. plebeius, C. baronessa and C. bennetti*) were found at sites with greater coverage of encrusting corals, massive corals, and leafy corals. Three species (*C. ephippium*, *C. trifascialis* and *C. auriga*) were found at sites with greater coverage of branching *Acropora*, bottlebrush *Acropora*, tabular *Acropora*, coral rubble and mushroom corals. One species (*C. lunulatus*) was found at sites with greater coverage of branching *Acropora*, encrusting corals, and massive corals.

### Feeding behavior

**Figure 4:**
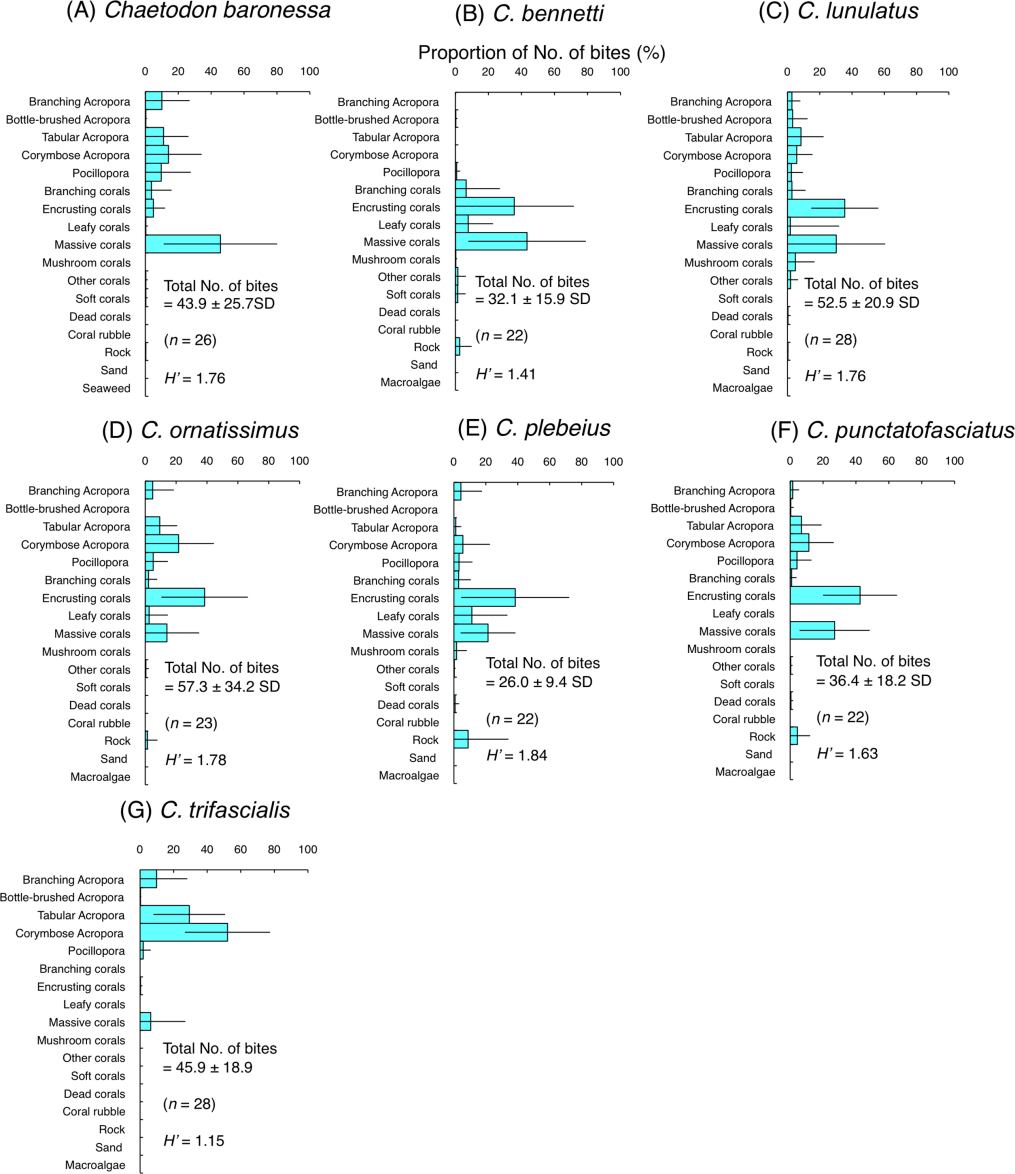
Average proportion of number of bites per 5 minutes for seven species of obligate coral polyp feeders. (A) *Chaetodon baronessa*, (B) *C. bennetti*, (C) *C. lunulatus*, (D) *C. omatissimus*, (E) *C. plebeius*, (F) *C. punctatofasciatus*, (G) *C. trifascialis*. The bars on each graph represent the standard deviation (SD). The total number of feeding bites ± SD, the total number of individuals (*n*) and the degree of substrate use diversity (*H’*) are also shown.

Among the seven species of obligate coral polyp feeders, five species had higher *H*’-values (1.63–1.84) indicating higher feeding plasticity, but selected different coral species. Most of the bites of *C. baronessa* were directed to tabular *Acropora*, corymbose *Acropora*, *Pocillopora* and massive corals (Fig. 4A). *C. lunulatus*, *C. plebeius*, and *C. punctatofasciatus* directed most of their bites to encrusting corals and massive corals (Figs. 4C, 4E and 4F). *C. ornatissimus* directed most of its bites toward corymbose *Acropora* and encrusting corals (Fig. 4D). In contrast, the remaining two species had lower *H*’-values (1.15–1.41) indicating lower feeding plasticity. *C. bennetti* directed most of the bites to encrusting corals and massive corals (Fig. 4B), whereas *C. trifascialis* directed most of the bites to tabular *Acropora* and corymbose *Acropora* (Fig. 4G).

**Figure 5:**
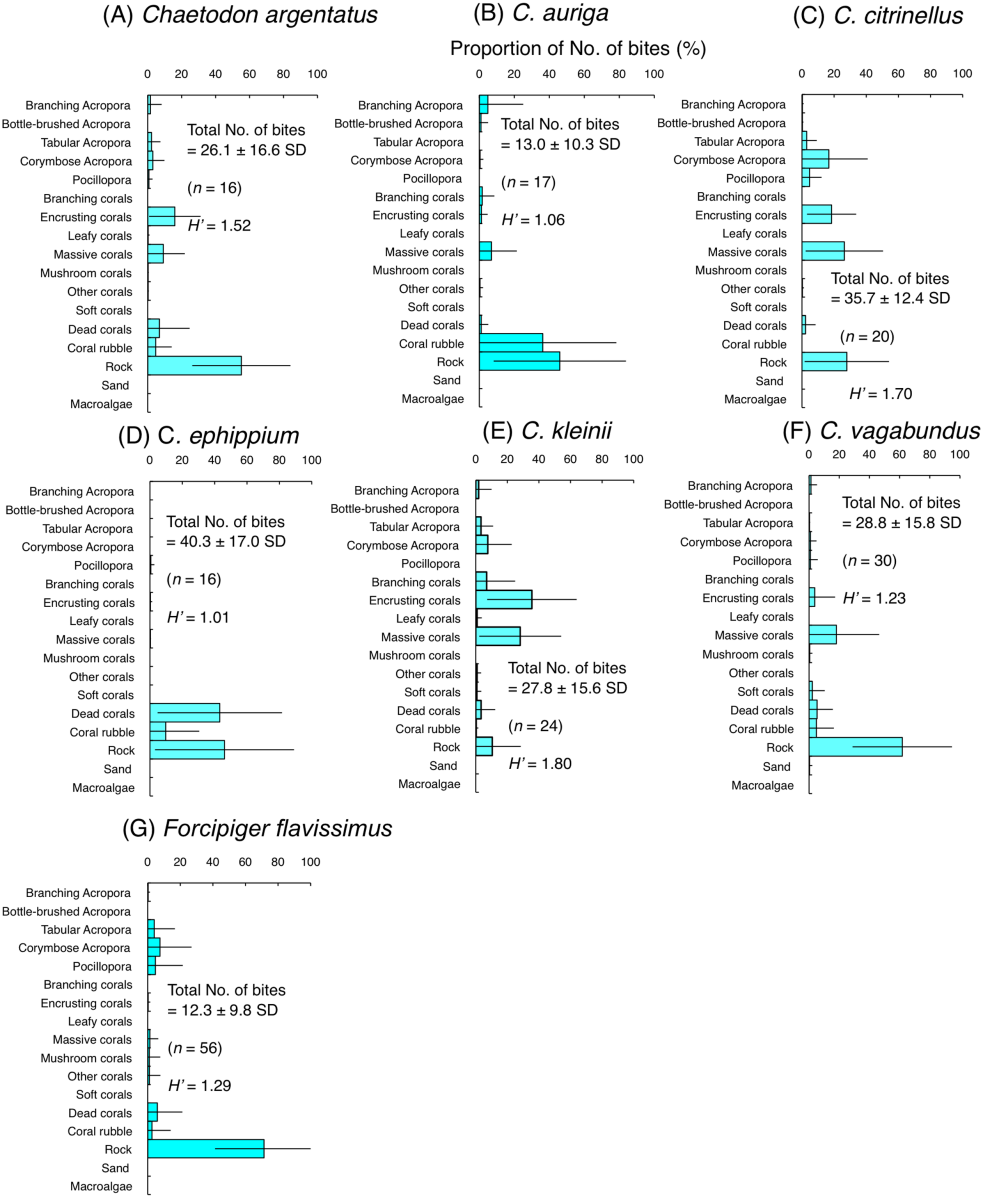
Average proportion of numbers of bites per 5 minutes for six species of facultative coral polyp feeders (A–F) and one species of non-coralline invertebrate feeder (G). The bars on each graph represent the standard deviation (SD). The total number of feeding bites ± SD, the total number of individuals (*n*) and the degree of substrate use diversity (*H’*) are also shown.

Among six species of facultative coral polyp feeders, three species (*C. argentatus*, *C. citrinellus* and *C. kleinii*) had relatively high *H*’-values (1.52–1.80), indicating high plasticity of substrate use for foraging. However, different coral species were selected among the three species. Most of the bites of *C. argentatus* were directed to encrusting corals, massive corals and rock (Fig. 5A). *C. citrinellus* directed most of its bites to corymbose *Acropora*, encrusting corals, massive corals and rock (Fig. 5C). *C. kleinii* directed most of its bites to encrusting corals and massive corals (Fig. 5E). In contrast, the remaining three species (*C. auriga*, *C. ephippium* and *C. vagabundus*) exhibited smaller *H*′-values (1.01–1.23), indicating relatively limited substrate use. *C. auriga* directed most of its bites towards coral rubble and rock (Fig. 5B). *C. ephippium* directed most of its bites to dead corals and rock (Fig. 5D). Most of bites of *C. argentatus* were directed to rock (Fig. F).

Non-coralline invertebrate feeder (*F. flavissimus*) exhibited smaller *H*’-value (1.29), indicating relatively limited substrate use. Most of the bites of *F. flavissimus* were directed to rock, but feeding bites were also notable on tabular *Acropora*, corymbose *Acropora* and *Pocillopora* (Fig. 5G).

### Substrate preference

**Figure 6:**
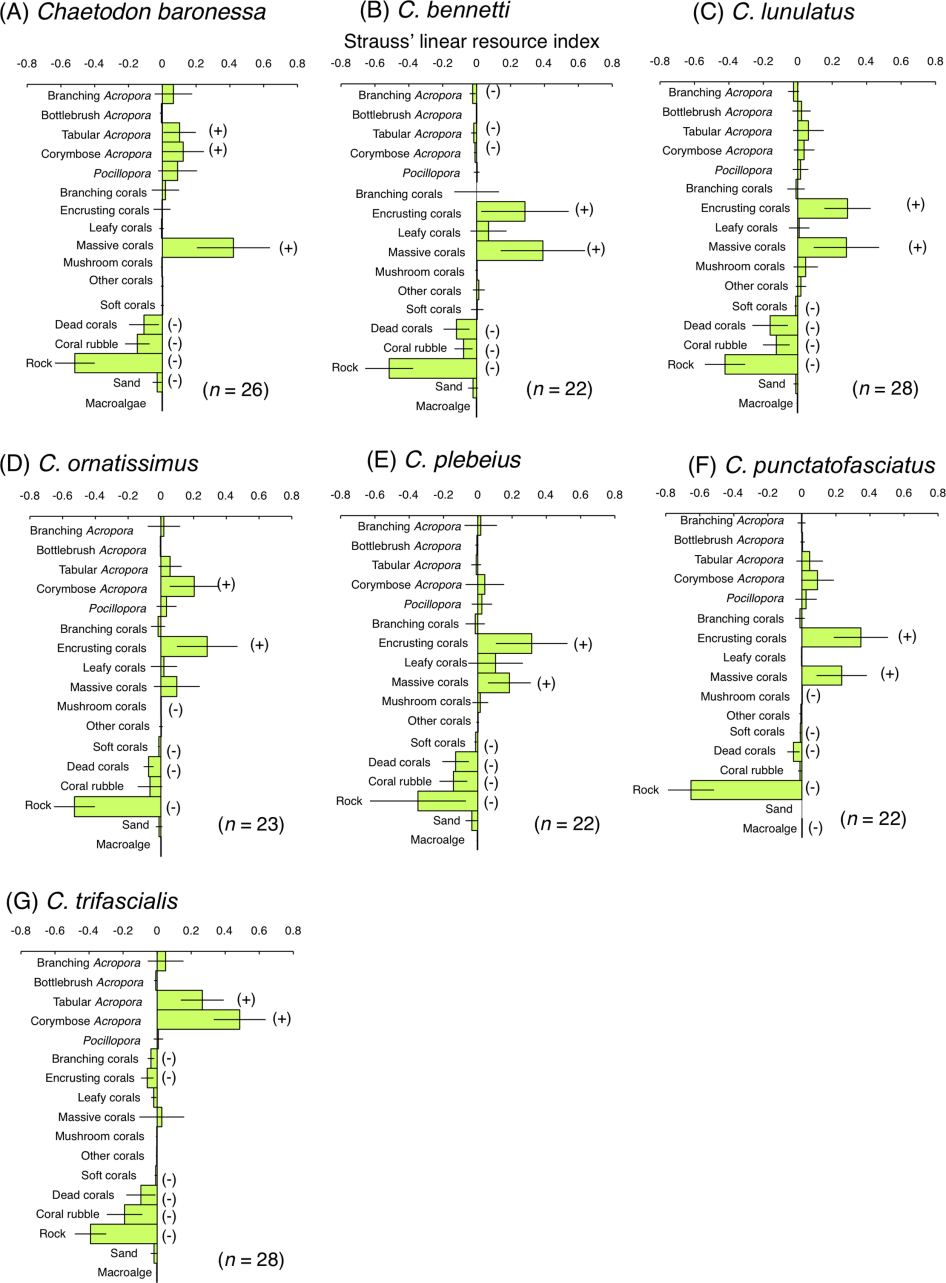
Strauss’ linear resource indices for seven species of obligate coral polyp feeder showing the feeding substrate selectvity regarding 17 substrates. (A) *Chaetodon baronessa*, (B) *C. bennetti*, (C) *C. lunulatus*, (D) *C. omatissimus*, (E) *C. plebeius*, (F) *C. punctatofasciatus*, (G) *C. trafascialis*. The bars on each graph represent 95% confidence intervals. Substrates with (+) and (−) represent significant positive use and negative use, respectively. The total number of individuals (*n*) is also shown.

Among obligate coral polyp feeders, *C. baronessa* exhibited significant positive use of tabular *Acropora*, corymbose *Acropora* and massive corals (Fig. 6A). Four species ( *C. bennetti*, *C. lunulatus*, *C. plebeius* and *C. punctatofasciatus*) showed significant positive use of encrusting corals and massive corals (Figs. 6B, 6C, 6E and 6F). *C. ornatissimus* showed significant positive use of corymbose *Acropora* and encrusting corals (Fig. 6D). In contrast, *C. trifascialis* showed significant positive use of tabular *Acropora* and corymbose *Acropora* (Fig. 6G). All seven species exhibited significant negative use of dead corals and rocks. Most species showed significant negative use of soft corals and coral rubble.

**Figure 7:**
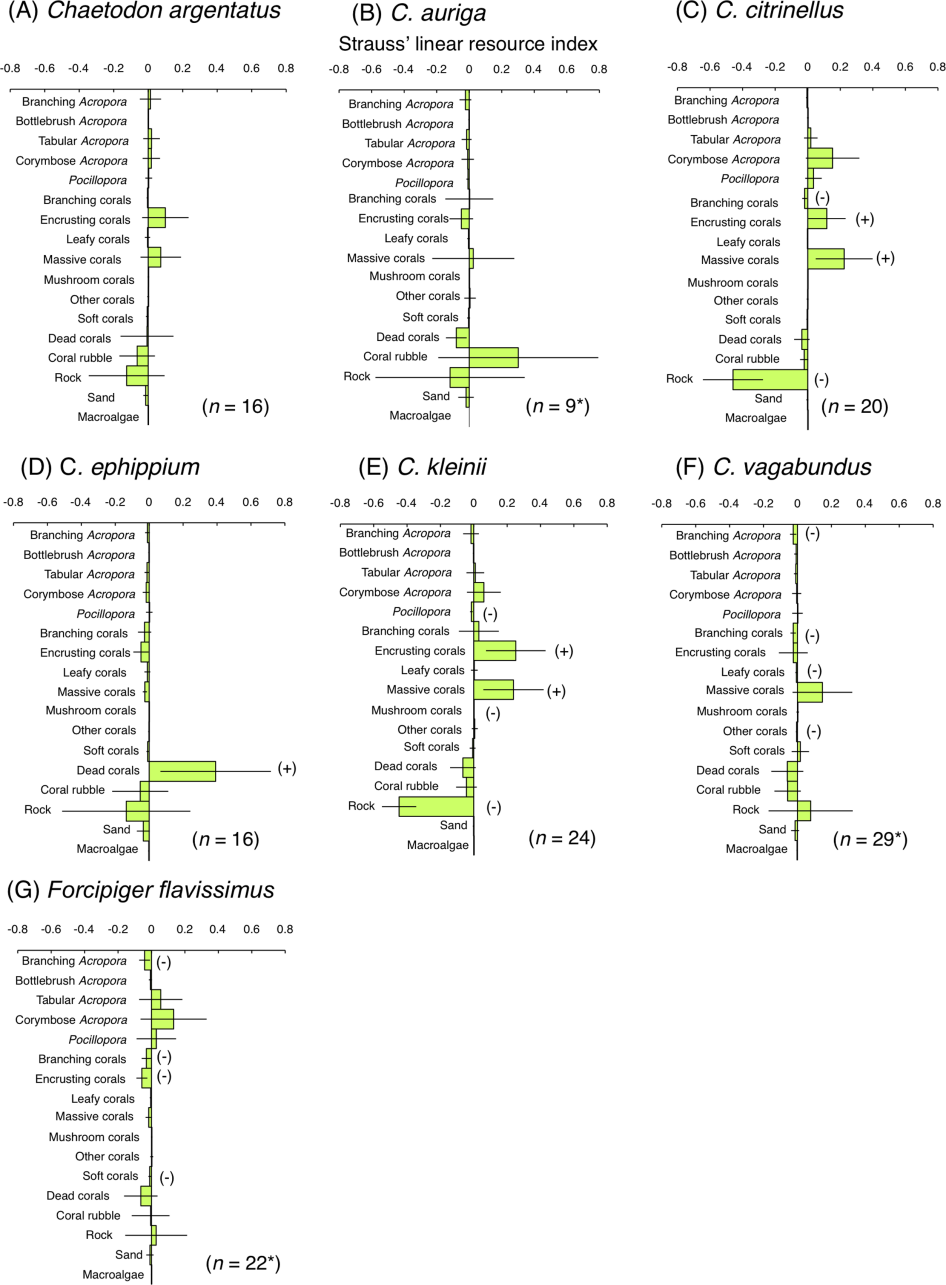
Strauss’ linear resource indices for six species of facultative coral polyp feeders (A–F) and of one non-coralline invertebrate feeder (G) showing the feeding substrate selectivity regarding 17 substrates. The bars on each graph represent 95% confidence intervals. Substrates with (+) and (−) represent significant positive use and negative use, respectively. The total number of individuals (*n*) is also shown. *: sample sizes are smaller than those in Fig. 5, because individuals whom total number of bite was less than 10 were omitted from the analysis.

For the facultative coral polyp feeders, three species (*C. argentatus*, *C. auriga* and *C. vagabundus*) showed no significant positive use of any substrate (Figs. 7A, 7B and 7F). *C. citrinellus* and *C. kleinii* showed significant positive use of encrusting corals and massive corals and significant negative use of rock (Figs. 7C and 7E). *C. ephippium* showed significant positive use of dead corals (Fig. 7D).

For the non-coralline invertebrate feeder (*F. flavissimus*), no significant positive use was found for any substrate (Fig. 7G). However, a positive tendency were found for tabular *Acropora* and corymbose *Acropora*.

### Overall trends in feeding behavior

**Figure 8:**
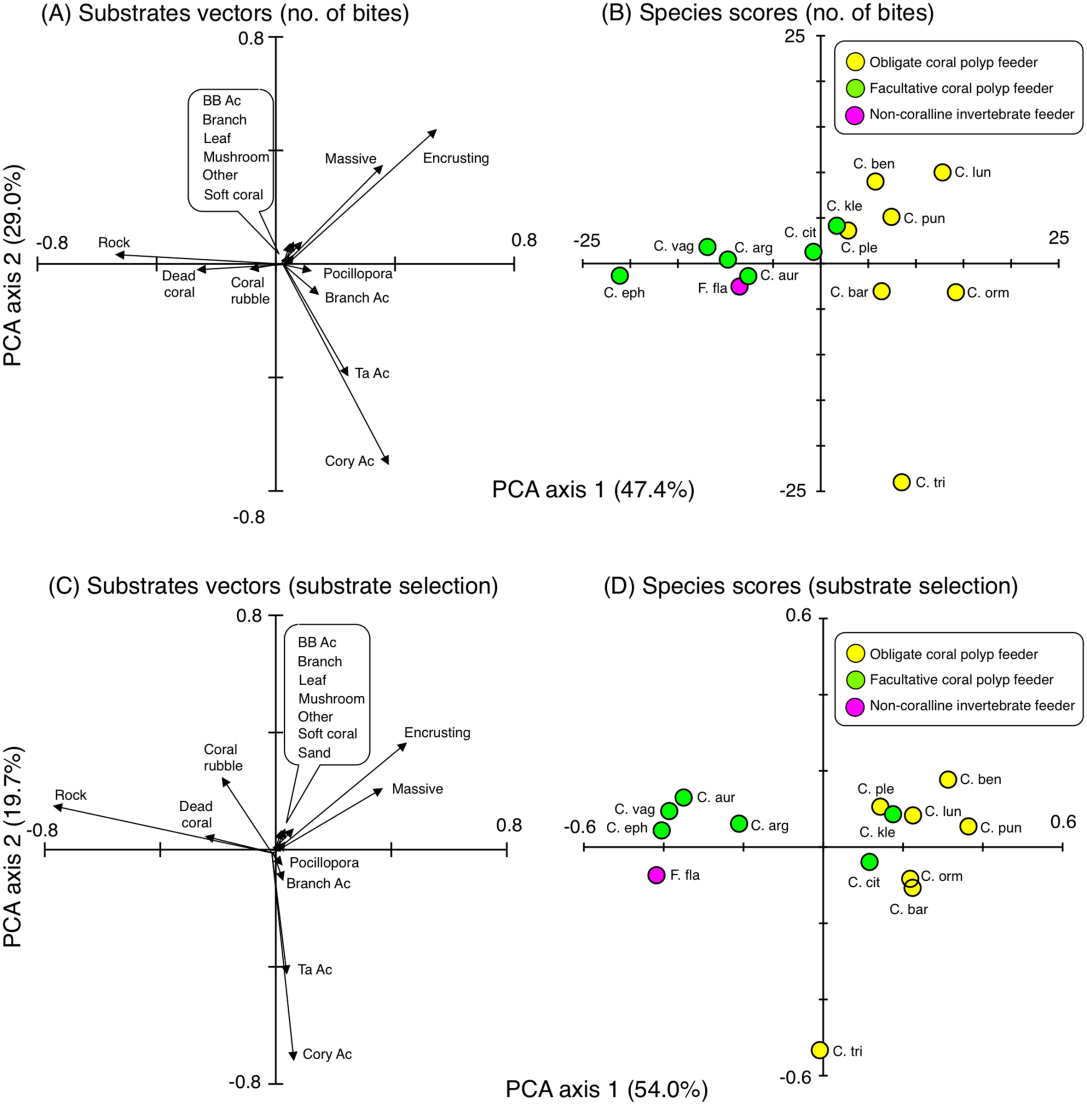
Results of principal component analysis (PCA) for feeding behavior showing the overall trends in feeding behavior of 14 butterflyfish species based on the numbers of bites (A, B) and Strauss’ linear resource index (C, D). In (A), since length of vectors for six substrates (bottlebrush Acropora, branching corals, leafy corals, mushroom corals, other corals and soft corals) were short, these substrates were indicated as markup ballon. In (C), since length of vectors for seven substrates (bottlebrush Acropora, branching corals, leafy corals, mushroom corals, other corals, soft corals and sand) were short, these substrates were indicated as markup ballon. The vectors for sand in (A) and for macroalgae in (A) and (C) are 0. In (A) and (C), some types of coral are represented as abbreviations (BB Ac: bottlebrush *Acropora*, Branch Ac: branching *Acropora*, Cory Ac: corymbose *Acropora*, TA Ac: tabular *Acropora*,). In (B) and (D), species names are represented as abbreviations (C. arg: *C. argentatus*, C. aur: *C. auriga*, C. bar: *C. baronessa*, C. ben: *C. bennetti*, C. cit: *C. citrinellus*, C. eph: *C. ephippium*, C. kle: *C. kleinii*, C. lun: *C. lunulatus*, C. orn: *C. ornatissimus*, C. ple: *C. plebeius*, C. pun: *C. punctatofasciatus*, C. tri: *C. trifascialis*, C. vag: *C. vagabundus*, F. fla: *Forcipiger flavissimus*). In (B) and (D), yellow, green and magenta symbols indicate obligate coral polyp feeder, facultative coral polyp feeder and non-coralline invertebrate feeder, respectively.

For the number of bites, PCA identified that six species (*C. bennetti*, *C. lunulatus*, *C. plebeius*, *C. punctatofasciatus*, *C. citrinellus* and *C. kleinii*) exhibited similar behavioral characteristics showing greater number of bites on encrusting corals and massive corals (Figs. 8A and 8B), whereas two species (*C. baronessa* and *C. ornatissimus*) directed most of their bites to encrusting corals or massive corals with somewhat frequent bites on branching *Acropora*, *Pocillopora*, corymbose *Acropora* and tabular *Acropora. C. trifascialis* directed most of its bites to corymbose *Acropora* and tabular *Acropora.* Five species (*C. argentatus*, *C. auriga*, *C. ephippium*, *C. vagabundus* and *F. flavissimus*) exhibited similar behavioral characteristics showing greater numbers of bites to rock, dead corals, and coral rubble.

For substrate selection, eight species (*C. bennetti*, *C. lunulatus*, *C. plebeius*, *C. punctatofasciatus*, *C. citrinellus, C. baronessa, C. ornatissimus* and *C. kleinii*) exhibited similar trends showing positive use of encrusting corals and massive corals (Figs. 8C and 8D). In contrast, five species (*C. argentatus*, *C. auriga*, *C. ephippium*, *C. vagabundus* and *F. flavissimus*) exhibited similar trends showing positive use of rock, dead corals and coral rubble. The trend of substrate selection of *C. trifascialis* was very different from the other 14 species, showing positive use of corymbose *Acropora* and tabular *Acropora.*

## Discussion

### Relationship between spatial distribution and feeding substrates

Some previous studies have shown the relationships between the spatial distribution of butterflyfish species and benthic communities at Great Barrier Reef. Three obligate coral polyp feeders (*C. baronessa*, *C. ornatissimus* and *C. plebeius*) were found at sites with greater coverage of acroporid corals (Pratchett & Berumen, 2008; Emslie et al., 2010). In contrast, few previous studies have shown the spatial distribution of two species of obligate coral polyp feeders (*C. bennetti* and *C. punctatofasciatus*). Thus, present study is the first study that revealed their spatial distributions. These five species were found at sites with greater coverage of encrusting corals, massive corals and rocks in the present study. The five species also exhibited greater proportion of bites on encrusting corals and massive corals. Thus, it is suggested that relatively close relationships between spatial distribution and feeding substrates were found for the five species. Since both encrusting corals and massive corals are less structurally complex, these corals are unlikely to provide refuges for these five species. In contrast, rocky surfaces inherently possess significant structural complexity (e.g., uneven surfaces and large holes). Thus, it is suggested that rocky areas with greater coverage of encrusting corals and massive corals may be suitable for these species as both habitat and foraging sites.

Emslie et al. (2010) showed that *C. lunulatus* was found at sites where various types of corals (Acroporidae, Faviidae, Musiidae, Pocilloporidae, Poritidae and Pectiniidae) were sympatrically found. Similar result was obtained in the present study, i.e., *C. lunulatus* was found at sites with greater coverage of encrusting corals, massive corals and branching *Acropora*. In the present study, *C. lunulatus* exhibited a greater proportion of feeding bites on encrusting corals and massive corals, but not on branching *Acropora*. Thus, *C. lunulatus* did not show a close relationship between spatial distribution and feeding substrate. Since branching *Acropora* has greater structural complexity, the corals can be suitable habitat for the species, rather than foraging substrate. Thus, among these three types of corals that affected the spatial distribution of *C. lunulatus*, the function might be different. Namely, branching *Acropora* is habitat and two types of corals (encrusting and massive corals) are foraging substrate, suggesting that sympatric distribution of these three types of corals would be suitable for this species.

At Great Barrier Reef, *C. trifascialis* was found at sites with greater coverage of tabular *Acropora* (Pratchett & Berumen, 2008; Emslie et al., 2010). In contrast, this species was found at sites with greater coverage of branching *Acropora* whereas exhibited greater proportion of bites on corymbose *Acropora* and tabular *Acropora* in the present study. This suggests the highly specialized nature of feeding, which has been shown to feed on a narrow range of corals (Berumen & Pratchett, 2008; Pratchett, 2005; Pratchett, 2007). A similar result was obtained at Lord Howe Island (Pratchett et al., 2014): a high density of *C. trifascialis* was encountered at sites with greater coverage of arborescent *Acropora*, whereas the species exhibited a significant preference for feeding on tabular *Acropora*. Thus, branching *Acropora* may serve as habitat whereas corymbose and tabular *Acropora* are suitable for foraging substrate for *C. trifascialis*, suggesting that the sympatric distribution of these three types of corals would be a suitable environment for this species.

For four facultative coral polyp feeders (*C. citrinellus*, *C. kleinii*, *C. vagabundus* and *C. argentatus*), *C. citrinellus* was found at sites with greater coverage of branching *Acropora* and isoporid corals whereas *C. kleinii* and *C. vagabundus* was found at greater coverage of non-acroporid corals (Pratchett & Berumen, 2008; Emslie et al., 2010). Since few studies clarified spatial distribution of *C. argentatus*, the present study is the first study that revealed the relationship between spatial distribution and foraging substrates for the species. These four species were found at sites with greater coverage of rock, that was different results from the previous studies at Great Barrier Reef. Since these four species exhibited greater proportion of bites on rock, encrusting corals and massive corals in the present study, rocky areas with greater coverage of encrusting corals and massive corals may be suitable for these species as both habitat and foraging sites. Remaining two species of facultative coral polyp feeders (*C. auriga* and *C. ephippium*) were found at sites with greater coverage of non-acroporid corals at Great Barrier Reef (Pratchett & Berumen, 2008; Emslie et al., 2010) whereas these species were found at the sites with greater coverage of branching *Acropora*, coral rubble and dead corals in the present study. Since *C. auriga* and *C. ephippium* respectively exhibited greater proportions of bites on coral rubble and dead corals, substrate characteristics would affect both the spatial distribution and foraging for these species.

The present study is the first study that clarified the spatial distribution of *Forcipiger flavissimus*, a non-coralline invertebrate feeder. The results suggest that there is a close relationship between spatial distribution and feeding substrates, since this species was found at sites with greater coverage of rock as well as exhibited greater proportion of bites on rock.

### Feeding substrates

Encrusting corals and massive corals were positively used by eight species (*C. baronessa*, *C. bennetti*, *C. lunulatus*, *C. ornatissimus*, *C. plebeius*, *C. punctatofasciatus*, *C. citrinellus* and *C. kleinii*) as feeding substrates. Corymbose *Acropora* and tabular *Acropora* were also positively used by three species (*C. baronessa*, *C. ornatissimus* and *C. trifascialis*). In contrast, no species of butterflyfishes exhibited a significant positive use of branching *Acropora* or bottlebrush *Acropora* in the present study. Pratchett et al. (2014) presented similar results, in which no significant positive use for arborescent *Acropora* was found among five species of butterflyfishes in Australia. An explanation for these findings may be found in the morphological characteristics of corals. The morphological complexity of four types of corals (encrusting corals, massive corals, corymbose *Acropora* and tabular *Acropora*) is less than that of branching and bottlebrush corals. It is suggested that butterflyfishes feed more easily on polyps of less complex corals than on corals with greater morphological complexity and this behavior is the reason why butterflyfishes prefer the above-mentioned four types of corals. Similar results are reported in previous studies (Tricas, 1989). Tricas (1989) showed that *C. multicinctus* preferred to feed on the massive coral, *Porites lobata,* rather than on the branching coral, *P. compressa*. Cole, Pratchett & Jones (2008) suggested that feeding efficiency is related to coral morphology. Thus, corals with lower morphological complexity (encrusting, massive, corymbose, and tabular corals) might be suitable foraging substrates on which butterflyfishes can easily feed.

Another explanation for coral preferences might be microstructural and nutritional characteristics of corals. Cole, Pratchett & Jones (2008) have suggested that polyp distribution, nematocyst size and lipid content could affect foraging substrate selection. For nutritional aspects, Pratchett (2013) has suggested that preferred corals by butterflyfishes as foraging substrate did not necessarily have greater nutrition among the various coral species. Quantitative comparison for such physical characteristics and nutrition among different coral species should be examined in the future.

In contrast, four species of facultative coral polyp feeders (*C. argentatus*, *C. auriga*, *C. ephippium* and *C. vagabundus*) exhibited greater numbers of bites on rock, although rock was not a significantly preferred substrate. Coral rubble and dead corals were also used as feeding substrates for *C. auriga* and *C. ephippium*, respectively. The diet for these species includes coral polyps and other benthic organisms, such as filamentous algae, polychaetes, sea anemones, hydroids, and sponges (Harmelin-Vivien & Bouchon-Navaro, 1983; Sano, Shimizu & Nose, 1984). Kramer, Bellwood & Bellwood (2014) demonstrated greater density of polychaetes in coral rubble and dead coral compared with living *Acropora* colonies. Thus, coral rubble and dead corals might be suitable feeding substrate.

One non-coralline invertebrate feeder (*F. flavissimus*) exhibited greater number of bites on rock. However, some of bites on tabular *Acropora*, corymbose *Acropora* and *Pocillopora* were also shown in the present study. The primary diet of this species consists of sponges, hydroids and polychaetes (Harmelin-Vivien & Bouchon-Navaro, 1983; Sano, Shimizu & Nose, 1984). As suggested by Cole & Pratchett (2013), this species might consume small invertebrates inhabiting corals. Further, Harmelin-Vivien & Bouchon-Navaro (1983) found small volume of scleractinians in the stomach contents of this species. Thus, although the species is categorized as invertebrate feeder, it might occasionally feed on coral polyps.

### Implications of coral bleaching

Coral bleaching has been frequently observed on Okinawan coral reefs and the decrease of living coral coverage and its negative effects on coral reef ecosystem have been concerning (e.g., Yara et al., 2009; Yara et al., 2014). In addition, coral bleaching due to global climate change is likely to increase on Okinawan coral reefs in the future (Okamoto, Nojima & Furushima, 2007; Yara et al., 2009; Hongo & Yamano, 2013). Susceptibility to coral bleaching varies among coral taxa in Okinawa as well as other regions (e.g., Fujioka, 1999; Yamazato, 1999; Marshall & Baird, 2000; Loya et al., 2001; Pratchett et al., 2013). Greater susceptibilities and higher rates of subsequent death have been reported for corals in the genera *Acropora* and *Pocillopora* (e.g., Loya et al., 2001; McClanahan et al., 2004). In contrast, greater tolerance and higher survival rates against coral bleaching have been reported for other coral taxa, such as members of the families Faviidae, Merulinidae, Mussidae, Poritidae, and Oculinidae (Fujioka, 1999; Yamazato, 1999; Marshall & Baird, 2000). These coral families mainly consist of encrusting and massive corals (Nishihira & Veron, 1995).

Thus, on Okinawan coral reefs, one species of obligate coral polyp feeder (*C. trifascialis*) would be negatively affected by coral bleaching for both aspects of habitat and foraging substrates since greater densities were found at areas dominated by branching *Acropora* and the species shows a significant positive use of tabular *Acropora* and corymbose *Acropora*. Pratchett et al. (2008) and Wilson, Graham & Pratchett (2013) reached a similar conclusion for this species, since the species is ecologically specialized to particular coral species. Wilson, Graham & Pratchett (2013) summarized the susceptibility of butterflyfishes to habitat disturbance and showed that *C. trifascialis* is the most vulnerable to living coral loss by bleaching.

On Okinawan coral reefs, feeding behavior of two obligate coral polyp feeders (*C. baronessa* and *C. ornatissimus*) would also be negatively affected by coral bleaching, since these species exhibited significant positive use of tabular *Acropora* and corymbose *Acropora.* However, the negative effect may not be severe since these two species inhabit areas with less coverage of acroporid corals and show positive feeding selectivity for massive corals or encrusting corals. In addition, three species (*C. lunulatus*, *C. ephippium* and *C. auriga*) would be negatively affected by habitat loss, since these three species inhabit sites with greater coverage of branching *Acropora*. If extensive coral loss by global bleaching events were seen, most of butterflyfish species might feed on less preferred substrates and expand foraging ranges to compensate the lost of their ecological requirements

Since the other eight species (*C. argentatus*, *C. bennetti*, *C. plebeius*, *C. punctatofasciatus*, *C. citrinellus*, *C. kleinii*, *C. vagabundus* and *F. flavissimus*) inhabit sites with greater coverage of rock, encrusting corals and massive corals, a major negative impact from coral bleaching may not occur on Okinawan coral reefs. However, since six species (*C. argentatus*, *C. plebeius*, *C. punctatofasciatus*, *C. citrinellus* and *C. kleinii*) exhibited feeding bites on acroporid corals, there is likely to be some negative effect. Based on the findings from the present study, the key corals for conservation would be branching *Acropora* as habitat and four types of coral (massive corals, encrusting corals, tabular *Acropora* and corymbose *Acropora*) as foraging substrates. However, as suggested by Pratchett et al. (2008), the long-term effects of coral loss causing less structural complexity should be carefully considered. Diverse coral communities should be conserved to maintain high species diversity of butterflyfish assemblages in Okinawan coral reefs.

## Conclusion

The present study revealed spatial distributions and feeding behavior of 14 species of butterflyfishes. It is suggested that various types of corals were primary determinants of spatial distribution. However, the effects of other ecological characteristics such as social behaviors as well as inter- and intra-specific competition on the spatial distribution should be considered. In addition, effects of other environmental variables (e.g., topographic complexity and wave exposure) on the spatial distribution should be examined. For foraging substrate selection, various type of living corals were positively selected as foraging substrate for all obligate coral polyp feeders and some facultative coral polyp feeders. However, the reason why these species selected particular types of corals is still unknown. In addition, some species, especially for facultative coral polyp feeders, may use living corals as foraging substrates, i.e., some species may feed on non-coral invertebrates inhabiting in coral colonies. Although the present study suggests that diverse coral species (especially encrusting corals, massive corals, tabular *Acropora*, corymbose *Acropora* and branching *Acropora*) should be conserved to maintain high species diversity of butterflyfishes in Okinawan region, it is important to know whether similar patterns occur in other regions.

##  Supplemental Information

10.7717/peerj.9666/supp-1 Figure S1 Spatial distributions of the 17 substrates White circles indicate coverage of substrates. The circle size represent the coverage. White crosses indicate no coverage. Each panel also shows whether or not the difference in density between exposed and inner reefs was statistically significant based on a GLMM (N.S. = non-significant: see also Tables S4 for details about results of GLMM).). Photo credit: International Coral Reef Research and Monitoring Center.

10.7717/peerj.9666/supp-2 Figure S2 Field data collection system A digital camera was attached with the data collection board by using PVC pipes.

10.7717/peerj.9666/supp-3 Table S1 List of butterflyfishes that are recorded at Okinawan coral reefs in accordance with Nakabo (2000) Species that were found at the 68 sites in the present study were shown at upper side of the table. The high density species for each dietary group (7 species of obligate coral polyp feeder, 6 species for facultative coral polyp feeder and 1 species for non-coralline invertebrate feeder) were selected for the analysis (indicated as +). Species that were not found at the 68 sites were also shown in the lower side of the table. For the species at lower side of the table, dietary group were not shown. Dietary group are represented as abbreviations: O, obligate coral polyp feeder; F, facultative coral polyp feeders; N, non-coralline invertebrate feeder, P, Plankton feeder.

10.7717/peerj.9666/supp-4 Table S2 Summary of dietary group for 14 butterflyfish species

10.7717/peerj.9666/supp-5 Table S3 Average number of individuals, standard deviations (SD) and results of generalized linear mixed model (GLMM) for 14 butterflyfish species *: significant difference after Bonferroni correction.

10.7717/peerj.9666/supp-6 Table S4 Average coverage, standard deviations (SD) and results of generalized linear mixed model (GLMM) for 17 substrates *: significant difference after Bonferroni correction.

10.7717/peerj.9666/supp-7 Supplemental Information 7 Raw data of Fig. S1

10.7717/peerj.9666/supp-8 Supplemental Information 8 Raw data of Fig. 2

10.7717/peerj.9666/supp-9 Supplemental Information 9 Raw data of Fig. 3

10.7717/peerj.9666/supp-10 Supplemental Information 10 Raw data of Fig. 4

10.7717/peerj.9666/supp-11 Supplemental Information 11 Raw data of Fig. 5

10.7717/peerj.9666/supp-12 Supplemental Information 12 Raw data of Fig. 6

10.7717/peerj.9666/supp-13 Supplemental Information 13 Raw data of Fig. 7

10.7717/peerj.9666/supp-14 Supplemental Information 14 Raw data of Fig. 8

## Additional Information and Declarations

### Data Availability

The following information was supplied regarding data availability: The raw measurements are available in the Supplemental Files.
