# Supplementary figures and images for: Spatial distribution and feeding substrate of butterflyfishes (family Chaetodontidae) on an Okinawan coral reef
Source: PeerJ. 2020 Aug 4;8:e9666. doi: 10.7717/peerj.9666 (PMC7413084; doi:10.7717/peerj.9666)

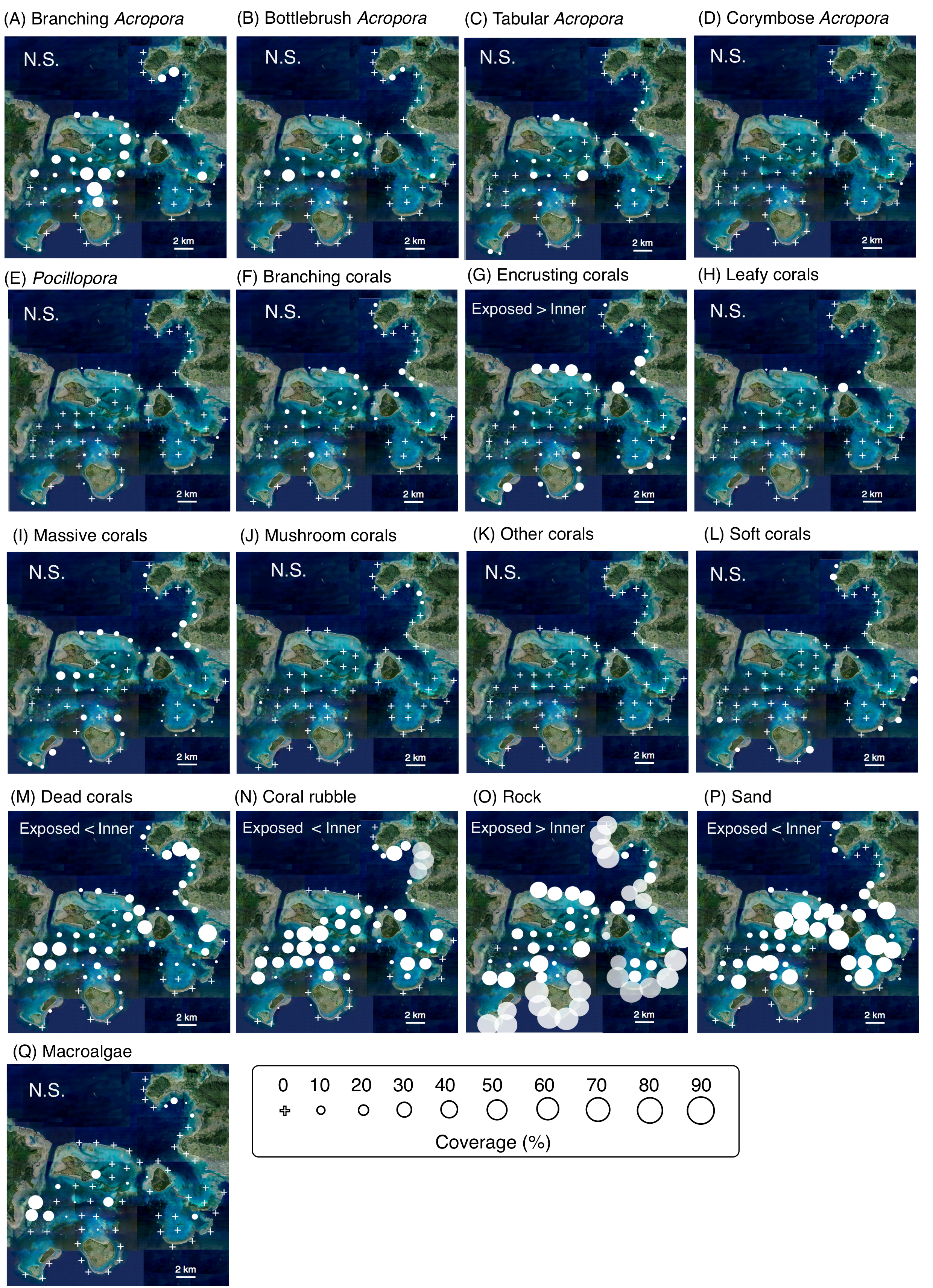

Supplement: Figure S1 — White circles indicate coverage of substrates. The circle size represent the coverage. White crosses indicate no coverage. Each panel also shows whether or not the difference in density between exposed and inner reefs was statistically significant based on a GLMM (N.S. = non-significant: see also Tables S4 for details about results of GLMM).). Photo credit: International Coral Reef Research and Monitoring Center. [file peerj-08-9666-s001.png]

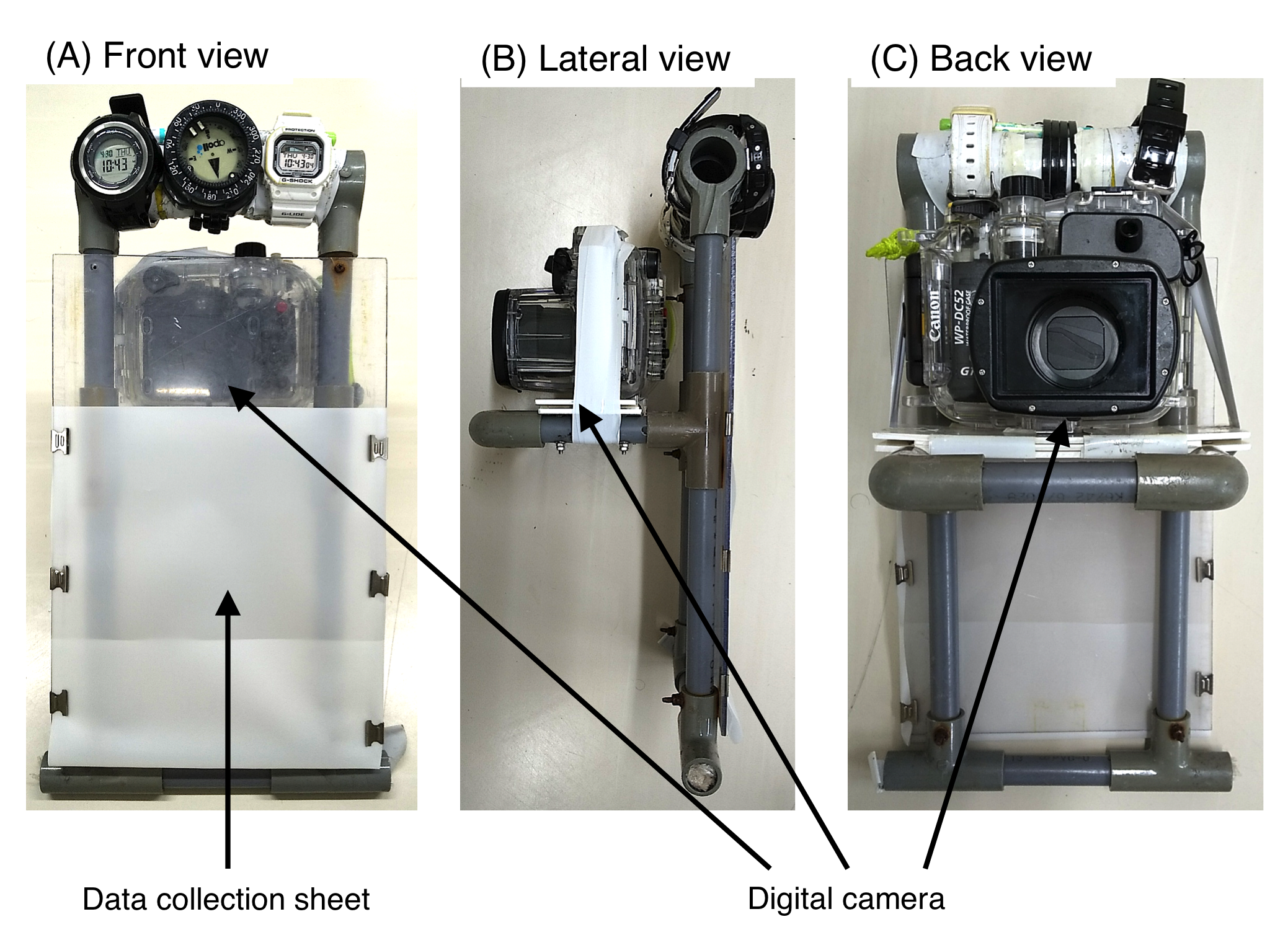

Supplement: Figure S2 — A digital camera was attached with the data collection board by using PVC pipes. [file peerj-08-9666-s002.png]
